# Expression of nitrous oxide reductase from *Pseudomonas stutzeri* in transgenic tobacco roots using the root-specific *rolD* promoter from *Agrobacterium rhizogenes*

**DOI:** 10.1002/ece3.74

**Published:** 2012-02

**Authors:** Shen Wan, Amanda M Johnson, Illimar Altosaar

**Affiliations:** Department of Biochemistry, Microbiology and Immunology, Center for Research on Environmental Microbiology – CREM, Faculty of Medicine, University of Ottawa451 Smyth Road, Ottawa, Ontario, K1H 8M5, Canada

**Keywords:** Greenhouse gas, nitrous oxide, nitrous oxide reductase, phytoremediation, rhizosecretion, root-specific expression

## Abstract

The nitrous oxide (N_2_O) reduction pathway from a soil bacterium, *Pseudomonas stutzeri*, was engineered in plants to reduce N_2_O emissions. As a proof of principle, transgenic plants expressing nitrous oxide reductase (N_2_OR) from *P. stutzeri*, encoded by the *nosZ* gene, and other transgenic plants expressing N_2_OR along with the more complete operon from *P. stutzeri*, encoded by *nosFLZDY*, were generated. Gene constructs were engineered under the control of a root-specific promoter and with a secretion signal peptide. Expression and rhizosecretion of the transgene protein were achieved, and N_2_OR from transgenic *Nicotiana tabacum* proved functional using the methyl viologen assay. Transgenic plant line 1.10 showed the highest specific activity of 16.7 µmol N_2_O reduced min^−1^ g^−1^ root protein. Another event, plant line 1.9, also demonstrated high specific activity of N_2_OR, 13.2 µmol N_2_O reduced min^−1^ g^−1^ root protein. The availability now of these transgenic seed stocks may enable canopy studies in field test plots to monitor whole rhizosphere N flux. By incorporating one bacterial gene into genetically modified organism (GMO) crops (e.g., cotton, corn, and soybean) in this way, it may be possible to reduce the atmospheric concentration of N_2_O that has continued to increase linearly (about 0.26% year^−1^) over the past half-century.

## Introduction

Of all the greenhouse gases, nitrous oxide (N_2_O) is the most damaging to the environment. With a global warming potential 310 times that of carbon dioxide, N_2_O contributes to the rising of atmospheric temperature (Ravishankara et al. 2009). N_2_O also causes acid rain as it reacts with the sun's ultraviolet rays and then ozone to form nitrate (Madigan et al. 2002).

Atmospheric levels of N_2_O have increased about 20% since preindustrial times (Richardson et al. 2009), and N_2_O continues to accumulate at a rate of 0.26% each year (Forster et al. 2007). The leading source of N_2_O is agricultural soil (Chapuis–Lardy et al. 2007). The amount of N_2_O produced is proportional to the amount of nitrogen that enters the soil (Crutzen et al. 2008). Nitrogen fertilizer consumption has increased from 31 million metric tonnes in preindustrial times to 165 million metric tonnes in 2005 (Burney et al. 2010) as a result of agricultural intensification. Thus, the increase in atmospheric N_2_O since preindustrial times is largely due to the application of nitrogen-based synthetic fertilizers to agricultural soils.

Nitrogen in the soil is metabolized by denitrifying bacteria such as *Pseudomonas stutzeri*, *P. aeruginosa*, *Bradyrhizobium japonicum*, and *Wolinella succinogenes* (Wunsch and Zumft 2005). A closer look into the denitrification process reveals five intermediates between fixable nitrogen and the inert atmospheric N_2_. The final step in denitrification is the enzymatic conversion of N_2_O to N_2_ by nitrous oxide reductase (N_2_OR). This reaction often does not occur if ideal metabolic conditions are not met, and results in emission of N_2_O (Zumft 1997). Furthermore, approximately a third of the denitrifying bacteria that have had their genomes sequenced have a truncated denitrification pathway, lacking the *nosZ* gene encoding the N_2_OR (Philippot et al. 2011). This last step of denitrification could become a core strategy for mitigating N_2_O emissions if crops could be improved with this agronomic trait.

The microbial N_2_OR is the only known biological catalyst that can catalyze the conversion of N_2_O to N_2_. The N_2_OR holoenzyme contains two identical subunits of 65.8 kDa, each containing six copper atoms. It catalyzes the copper-dependent two-electron reduction of N_2_O to water and dinitrogen gas, which takes place in the bacterial periplasm (Pomowski et al. 2011). In *P. stutzeri*, N_2_OR is encoded by the gene *nosZ* (Zumft 1997). The complete *nos* operon contains five additional *nos* genes, *nosR*, *nosD*, *nosF*, *nosY*, and *nosL*, each of which encodes proteins that are thought to assist in the assembly of the enzyme in *P. stutzeri*. *NosR* encodes a transcriptional regulator, *nosD*, *nosF*, and *nosY* encode an ABC-type transporter, and *nosL* encodes a copper chaperone (Honisch and Zumft 2003).

Here, we present a means of mimicking bacterial denitrification in plants by endowing them with the recombinant N_2_OR enzyme. This is a novel method of phytoremediation since, to our knowledge, no one has used plants as a means of mitigating this particular greenhouse gas at its source in the soil.

Plant roots are in direct contact with the microbial community in the rhizosphere. They secrete a number of chemicals into the rhizosphere, having a large impact on soil chemistry (Philippot et al. 2009). Tobacco plant roots have been used as a recombinant protein production system using root-specific promoters for the gene of interest (Drake et al. 2003). Promoting complete denitrification in the rhizosphere in this way may eliminate N_2_O emissions at the source. The substrate, N_2_O, produced by denitrifiers in the rhizosphere could potentially bind to the catalytic enzyme, N_2_OR, secreted by transgenic tobacco plant roots. N_2_O reduction would occur, resulting in the release of N_2_ gas into the soil air pockets and ultimately into the atmosphere. To test this hypothesis, in an attempt to achieve N_2_OR expression *in planta*, *Nicotiana tabacum* cv. Xanthi nc. plants were transformed with the single *nosZ* gene. A second set of transgenic plants were also transformed with the more complete operon, *nosFLZDY*. Transgenic plants were analyzed to confirm transgene incorporation, transgene expression, protein expression, and protein activity. Surprisingly, extracts from these tobacco plants, isolated from their root tissue and from the medium surrounding their roots, when analyzed by the methyl viologen assay provided evidence of N_2_O reduction capacity.

## Materials and Methods

### Genomic DNA isolation from *P. stutzeri*

*Pseudomonas stutzeri* Zobell (ATCC 14405) cells were plated on Luria broth (LB)-agar medium and grown at 30°C for 48 h. A single colony was used to inoculate 5 mL of liquid LB medium, and mixture was incubated at 30°C overnight with shaking. The bacterial culture was added to 100 mL liquid LB medium and incubated for 3 h. Methods of Neumann et al. (1992) were followed for extraction of genomic DNA. A total of 100 mL bacterial culture was centrifuged at 3000 *g* for 15 min at 4°C, the pellet was washed in phosphate buffered saline (PBS) buffer, and resuspended in 5 mL SET (75 mM NaCl, 25 mM EDTA, 20 mM Tris pH 7.5). Lysozyme was added to a concentration of 1 mg mL^−1^, and the resulting suspension incubated at 37°C for 30 min, mixing occasionally by inversion. To inactivate DNases, 0.5 mL of proteinase K (1 mg mL^−1^) was added along with 0.5 mL 10% sodium dodecyl sulfate (SDS), and mixture was incubated at 55°C for 2 h with occasional inversion.

To precipitate proteins out of solution, 2.5 mL of 5 M NaCl was added and gently mixed. Adding 10 mL chloroform, the tube was mixed at low speed for 1 h, and then centrifuged at 3000 *g* for 10 min. The aqueous upper phase was transferred to a new tube. Introduction of 20 mL isopropanol, mixing by inversion, and incubation at –20°C for 15 min were sufficient to precipitate DNA. The DNA was wound onto a pipette tip, washed twice with 70% ethanol in a new 1.5-mL tube, placed in a centrifugal evaporator to dry, and resuspended in 500 µL deionized water.

### Engineering of plant expression constructs

*Pseudomonas stutzeri* genomic DNA was used as a template for polymerase chain reaction (PCR) amplification of the *nosZ* gene or *nosFLZDY* genes. Primers were gene-specific, and were designed to introduce restriction sites so the resulting segments could be fused (Table S1). The root-specific promoter of the *rolD* gene from *Agrobacterium rhizogenes* was chosen to direct root-specific expression of N_2_OR. The promoter sequence 426D (Elmayan and Tepfer 1995), containing a 463 bp segment of the upstream untranscribed region of *rolD*, was amplified from the plasmid pLJ1 provided by D. Tepfer. The nopaline synthase polyadenylation sequence was used as terminator (NOSter). The leader sequence of the *Daucus carota* (carrot) *extensin* (ex) gene was amplified from the pHBV-CO plasmid using forward primer, ExSigZF/ExSigDF, and reverse primer, ExSigZR/ExSigDR (Chen and Varner 1985; Alli et al. 2002). The *Phaseolus vulgaris* (*Pv*) alpha amylase inhibitor-1 signal sequence was amplified using forward and reverse primers PvSigF and PvSigR, respectively (Prescott et al. 2005). Purified PCR products were cloned into the pPCR-Script Amp SK (+) cloning vector system (Stratagene, La Jolla, CA). For assembly of expression vector prolD-*nosZ*, segments of NOSter, nosZ, ex, and rolD were ligated together and cloned into the binary vector pRD400 (Datla et al. 1992) (Fig. S1) following digestion with *Kpn*I and *Bam*HI. Plasmid pRD400 carries the neomycin phosphotransferase II (*nptII*) gene, which confers resistance to the antibiotic kanamycin. The resulting prolD-*nosZ* plasmid was sequenced at StemCore Laboratories (Ottawa, Canada) to verify accuracy of the DNA sequence. The megacassette prolD-*nosFLZDY* was constructed in a similar fashion, ligating individual expression constructs for *nosF*, *nosD*, *nosY*, and *nosL* and cloning the resulting sequence into pRD400 (Fig. S2).

### Plant transformation and selection

*Agrobacterium tumefaciens* strain LBA4404 was introduced with plasmids prolD-*nosZ* and prolD-*nosFLZDY.* Tobacco (*N. tabacum* cv. Xanthi) seeds were sterilized with a 1-min wash in 70% ethanol, 10-min wash in 10% bleach containing 1–2 drops Tween-20, and three rinses in sterile distilled water. Sterilized seeds were grown on germination medium (1/2 MS, 3% sucrose, 0.8% agar, pH 5.8) in petri dishes. Tobacco leaf transformation followed the general method of Horsch et al. (1985). Leaves were collected from 5- to 6-week-old plants and cut into sections under sterile conditions. *Agrobacterium tumefaciens* harboring either prolD-*nosZ* or prolD-*nosFLZDY* plasmid was used to infect leaf sections for 48 h on cocultivation medium (MS, 3% sucrose, 0.8% agar, 1.0 mg L^−1^ 6-benzyladenine, 0.1 mg L^−1^α-naphthalene acetic acid, pH 5.8).

Transformed leaf cells were grown on regeneration medium (MS, 3% sucrose, 0.8% agar, 1.0 mg L^−1^ 6-benzyladenine, 0.1 mg L^−1^α-naphthalene acetic acid, pH 5.8, 200 mg L^−1^ of ticarcillin, and 300 mg L^−1^ of kanamycin). Calli began to appear after two weeks. When the shoots had reached a height of 1 cm, they were excised and transferred to root generating medium (MS, 2% sucrose, 0.8% agar, pH 5.8, 200 mg L^−1^ of ticarcillin, and 300 mg L^−1^ of kanamycin). When the roots had reached 5 cm long, the plantlets were transferred to potted soil and grew in the greenhouse where they were maintained through seed set with 16 h daylight (400 W/m^2^) at 25°C and 8 h dark at 21°C, with watering as necessary. Seeds were collected once seedpods had matured and planted in hydroponic medium to perform the rhizosecreted protein experiments described below.

### Polymerase chain reaction

A portion of the root tissue (100 mg) was collected from each 6-week-old plant and genomic DNA was extracted using the DNeasy Plant Mini Kit (Qiagen, Mississauga, Canada). PCR was performed in a 25 µL reaction mixture containing 50 ng of tobacco genomic DNA, 25 pmol of each primer, 2.5 µL of 10 × PCR buffer (with 1.5 mM MgCl_2_), 250 µM of each dNTP, 0.5 unit Taq DNA polymerase, with the following cycling condition: initial denaturation at 94°C for 4 min; 30 cycles with denaturation at 94°C for 30 sec, annealing at 55°C for 1 min, and elongation at 72°C for 2 min; followed by a final elongation at 72°C for 10 min. DNA from nontransgenic (NT) plant roots was used as negative control, while either the prolD-*nosZ* or prolD-*nosFLZDY* plasmid was used as positive control. The following primers were used to detect the *nosZ* gene in genomic DNA from prolD-*nosZ* transgenic plants: forward primer (ExSigZF) 5′-TAGGTACCTACTCGAGATGGGAAGAATTGCTAGAGG-3′ and reverse primer (NosZR) 5′-TAGGATCCAACATATGTTAGGCCGGCTCGACCATC-3′. For prolD-*nosFLZDY* transgenic plants, the specific primers used to detect the *nosZ* gene were forward primer (NosZF) 5′-GACTAGTCAGGCCGTCAAGGAGTCCAAG-3′ and reverse primer (NosZR) 5′-TAGGATCCAACATATGTTAGGCCGGCTCGACCATC-3′.

### Reverse transcription PCR

Root tissue (100 mg) was collected from each 6-week-old plant and total RNA was extracted using the RNeasy Plant Mini Kit (Qiagen). After treatment with RNase-free DNase (Promega, Madison, WI), RT-PCR was performed according to the SuperScript II Reverse Transcriptase system protocol (Invitrogen, Carlsbad, CA). In a 10 µL reaction mixture, 250 ng of total RNA was reverse transcribed at 70°C for 10 min with an oligo (dT) primer. The conditions for cDNA synthesis were 10 min at 25°C, 60 min at 37°C, and 5 min at 95°C. Each PCR reaction used 2 µL cDNA as a template and *nosZ-*specific primers. DNA from NT plant roots was used as negative control, while either the prolD-*nosZ* or prolD-*nosFLZDY* plasmid was used as positive control. For prolD-*nosZ* transgenic plants, the following primers were used to detect the *nosZ* gene: forward primer (ExSigZF) 5′-TAGGTACCTACTCGAGATGGGAAGAATTGCTAGAGG-3′ and reverse primer (NosZR) 5′-TAGGATCCAACATATGTTAGGCCGGCTCGACCATC-3′. For prolD-*nosFLZDY* transgenic plants, the following primers were used to detect the *nosZ* gene: forward primer (NosZF) 5′-GACTAGTCAGGCCGTCAAGGAGTCCAAG-3′ and reverse primer (NosZR) 5′-TAGGATCCAACATATGTTAGGCCGGCTCGACCATC-3′. A control PCR, with total RNA not treated with reverse transcriptase, was performed to ensure that no DNA was present. Expression of other genes in the *nos* cassette (*nosF*, *no*s*L*, *nosD*, and *nosY*) was also analyzed using RT-PCR in the same way, with gene-specific primers.

### Growth of transgenic plants in hydroponic medium

Hydroponic cultures were established for the harvest of recombinant protein from root tissue and the rhizosphere according to the method of Drake et al. (2003). Transgenic tobacco seeds containing either the rolD-*nosZ* or the rolD-*FLZDY* expression cassette were surface-sterilized using 20% v/v bleach, washed in sterile distilled water, and sown onto a 9-cm petri dish containing 0.7% w/v agar-solidified MS medium (Murashige and Skoog 1962). When seedlings reached 1 cm in height, they were transferred to liquid MS medium by placing the shoot through a perforation in a plastic platform in a 50-mL container containing 25 mL liquid MS medium. The roots of the seedling were immersed in the liquid medium and the shoots were supported above the platform. Seedlings were maintained at 25°C with 16 h daylight and 8 h dark, and were grown to a height of 6 cm before further analysis.

### Crude protein extraction from roots and from hydroponic medium

Root tissue was harvested, washed three times in ddH_2_O, immediately frozen in liquid nitrogen, and ground to a fine powder using a mortar and pestle. The soluble protein was extracted from root tissue of transgenic tobacco using Plant Total Protein Extraction Kit (Sigma, St. Louis, MO). Total protein extract was quantified using the BCA Protein Assay Kit (Pierce Biotechnology Inc., Rockford, IL).

Rhizosecreted protein from each plant culture was collected over a period of 25 days. Aliquots of MS medium (500 µL) from both rolD-transgenic and NT plant cultures were collected at regular intervals. Samples were concentrated to a volume of 20 µL by means of YM50 Microcon filters (Millipore, Billerica, MA) and stored at –20°C. Protein samples were pooled together prior to western blotting and N_2_OR activity analysis.

### Anaerobic purification of N_2_OR from *P. stutzeri*

*Pseudomonas stutzeri* Zobell (ATCC 14405) cells were plated on LB-agar medium and grown at 30°C for 48 h. A single colony was used to inoculate 200 mL of synthetic medium (Matsubara et al. 1982), and culture was incubated at 30°C for 24 h with low-speed shaking. This culture was added to 1.8 L of synthetic medium and grown for 6 h. To induce N_2_OR expression, sodium nitrate was added to a concentration of 1 g L^−1^. The culture was scaled up to 12 L and incubated at 30°C for 24 h. After centrifugation, the resulting pellet was washed with 50 mM MgCl_2_ in 25 mM Tris buffer (pH 7.5), purged with argon, and stored at –70°C. After dissolving the pellet in 25 mM Tris buffer (pH 7.5), cells were disrupted by sonication.

The supernatant was applied to a DEAE Sepharose ion-exchange chromatography column and eluted at 2 mL min^−1^ using 25 mM Tris (pH 7.5) for buffer A, and 25 mM Tris (pH 7.5), 0.3 M NaCl for buffer B. Protein was eluted at 4°C over five column volumes with a linear gradient of 0–100% buffer B. Collected 5 mL fractions were stored at 4°C after purging with argon. Those containing N_2_OR and nitrite reductase were identified using sodium dodecyl sulfate polyacrylamide gel electrophoresis (SDS-PAGE), pooled and then dialyzed using a membrane with a 14-kDa cutoff. Fractions were subjected to hydroxyapatite chromatography using 10 mM NaH_2_PO_4_ (pH 7.2) for buffer A and 400 mM NaH_2_PO_4_ (pH 7.2) for buffer B. Protein was eluted over 10 column volumes with a linear gradient of 0–80% buffer B. Collected 2 mL fractions were purged with argon and stored at 4°C prior to SDS-PAGE analysis (Fig. S3). Chromatography was performed under anaerobic conditions, with all reagents degassed by vacuum and procedures carried out under 100% argon. Concentration of purified N_2_OR was determined using the Bradford method (1976) with bovine serum albumin as the standard.

### Western blotting

Samples were prepared by heating 40 µg protein extract for 10 min at 95°C in 2× protein sample buffer (0.1 M TrisCl pH 6.8, 1 mM EDTA, 6% SDS, 20% glycerol, 0.1% bromophenol blue, 5%β-mercaptoethanol). The positive control was N_2_OR purified from *P. stutzeri*, and the negative control was protein extract from a nontransformed plant. Samples were run on a 10% acrylamide gel with a 5% stacking gel, then transferred onto a nitrocellulose membrane using the Trans-Blot transfer cell (Bio-Rad, Hercules, CA). To prevent nonspecific binding, membrane was blocked with Tris-buffered saline with Tween (TBST) plus 5% skim milk before incubation with rabbit anti-N_2_OR serum (1:5000, provided by W. Zumft) and subsequently antirabbit biotin horseradish peroxidase-linked antibody (1:1000 in TBST) (Cell Signaling Technology, Danvers, MA). Immunoblotted N_2_OR was visualized using enhanced chemiluminescence (ECL) western blotting chemiluminescent reagents (Amersham Biosciences, Baie d’Urfé, QC).

### Methyl viologen-linked reductase activity assay

In vitro N_2_OR activity was determined using a modified protocol from Kristjansson and Hollocher (1980). This assay uses reduced methyl viologen as the chemical electron donor, allowing the reduction of N_2_O to be monitored spectrophotometrically. Assays were carried out in an anaerobic chamber (10% H_2_, 5% CO_2_, and 85% N_2_; Model 1025, Thermo Fisher Scientific, Waltham, MA) at 37°C, and all reagents were degassed by vacuum and purged with 100% argon gas. For the positive control, the reaction mixture comprised 5 µL purified N_2_OR (0.956 µg/µL), 0.05 mL of 10 mM methyl viologen, and 0.05 mL of 5 mM sodium dithionite in 2 mL of 10 mM KH_2_PO_4_ (pH 7.1). For transgenic root samples, the mixture comprised 50 µL root extract, 0.2 mL of 10 mM methyl viologen, and 0.2 mL of 5 mM sodium dithionite in 1.5 mL of 10 mM KH_2_PO_4_ (pH 7.1). For all samples, reactions took place in a stoppered 3.5-mL cuvette (light-path length 1 cm).

Absorbance at 600 nm was monitored for 1 min to quantify the background oxidation rate. The substrate, N_2_O, was added in one 25-µL injection of N_2_O-saturated ddH_2_O. By monitoring the change in absorbance at 600 nm of the reaction mixture, the specific activity of the N_2_OR therein was determined (for specific activity calculations, see Supplementary Methods). The specific activity was expressed as micromoles of N_2_O reduced per min per milligram of N_2_OR protein.

## Results

### Engineering and growth of nosZ-expressing tobacco plants

We generated transgenic *N. tabacum* cv. Xanthi nc. plants expressing the *nosZ* gene under the transcriptional control of the root-specific *A. rhizogenes rolD* promoter (Slightom et al. 1986). Fourteen kanamycin-resistant T_0_ *nosZ*-transgenic tobacco lines and 15 kanamycin-resistant T_0_ *nosFLZDY*-transgenic tobacco lines were generated by leaf-disk *Agrobacterium*-mediated transformation. The transgenic tobacco lines were designated rolD:*nosZ*-1.1 to rolD:*nosZ*-1.14, and rolD:*nosFLZDY*-5.1 to rolD:*nosFLZDY*-5.15. First generation transformed (T_1_) plant lines were grown from the seeds of the T_0_ generation, with no apparent phenotypic differences between transgenic and nontransgenic plants.

### Detection of *nosZ* DNA in transgenic plants

**Figure 1:**
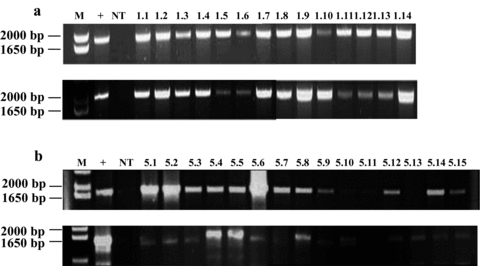
Identification of rolD-*nosZ* and rolD-*nosFLZDY* transgenic tobacco plant lines by PCR and RT-PCR screening. (A) Amplification of the 1869 bp *ex-nosZ* coding sequence in rolD-*nosZ* transgenic tobacco plants was detected by PCR (top row) and RT-PCR (bottom row). (B) Amplification of the 1767 bp *nosZ* coding sequence in rolD-*nosFLZDY* transgenic tobacco plants was detected by PCR (top row) and RT-PCR (bottom row). M = 1 kb Plus DNA ladder (Invitrogen); + = positive control, recombinant plasmid prolD-*nosZ*/prolD-*nosFLZDY* isolated from *Escherichia coli*; NT = negative control, was genomic DNA from a nontransformed plant. Transformed plants are identified by number, with 1-series plants representing those expressing rolD-*nosZ* and 5-series plants representing those expressing rolD-*nosFLZDY*.

To confirm the presence of the *nosZ* transgene under the control of the *rolD* promoter, transgenic plants were screened by PCR using sequence-specific primers. NT plants were used as negative controls, while the plasmid prolD-*nosZ* or prolD-*nosFLZDY* was used as a positive control. PCR analysis of the putative prolD-*nosZ* transformed lines showed the presence of a 1869-bp band (Fig. 1A, top row) corresponding to the length of the *ex*-*nosZ* coding sequence. For putative prolD-*nosFLZDY* transformed lines, PCR analysis was performed to amplify the 1767 bp *nosZ* coding sequence (Fig. 1B, top row), as well as fragments from *nosF*, *nosL*, *nosD*, and *nosY* (data not shown). PCR analysis confirmed the presence of *nosZ* in all transgenic lines, whereas no amplification was observed in NT lines.

### Detection of *nosZ* mRNA in transgenic plants

To confirm the transcription of the *nosZ* transgene under the control of the *rolD* promoter, RT-PCR was conducted to amplify the *nosZ* fragment of the transgene with gene-specific primer sets. RT-PCR products with the expected size of 1869 bp for *nosZ* mRNA were observed in all *nosZ*-transformed and 1767 bp for *nosZ* mRNA were observed in *nosFLZDY*-transformed transgenic lines (Fig. 1A and 1B, bottom row). In addition, RT-PCR fragments of the expected size for *nosF*, *nosL*, *nosD*, and *nosY* were produced from the rolD-*nosFLZDY* transgenic line (data not shown). NT controls did not exhibit any bands corresponding to *nosZ* transcripts. There were notable differences in transcript abundance among the *nosZ* and *nosFLZDY* transgenic lines. Transcript abundance was much higher in *nosZ*-transgenic plants than in *nosFLZDY*-transgenic plants. PCR was also conducted with total RNA not treated with reverse transcriptase to verify that there was no DNA contamination (data not shown).

### Protein expression analysis of root tissue by Western immunoblot

**Figure 2:**
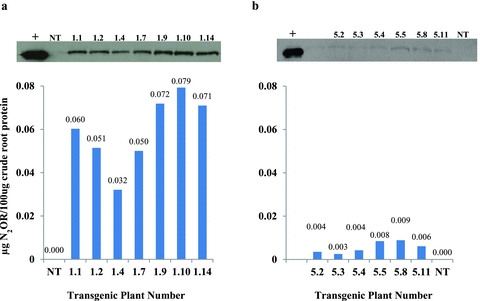
Western immunoblot analysis detecting N_2_OR in total soluble protein extracted from transgenic plant root tissue. (A) Histogram showing the yield of recombinant N_2_OR in *nosZ*-expressing transgenic plants, with the corresponding Western immunoblot. (B) Histogram showing the yield of recombinant N_2_OR in *nosFLZDY*-expressing transgenic plants, with the corresponding Western immunoblot. + = positive control, N_2_OR protein purified from *P. stutzeri*; NT = negative control, protein extracted from a nontransformed plant; transformed plants are identified by number.

To demonstrate the expression of the N_2_OR in tobacco root from T_0_ transgenic plants, an immunoblot assay of total soluble protein extracts was performed using a polyclonal antibody against the N_2_OR antigen. Fig. 2 displays the immunoblots for rolD-*nosZ* and rolD-*nosFLZDY* samples, showing a single band of the expected molecular mass of 72 kDa. The positive control, N_2_OR obtained from *P. stutzeri*, also yielded a signal band at 72 kDa. There was no signal for the presence of N_2_OR in nontransformed plant extracts. The images were scanned using ImageJ software (http://rsbweb.nih.gov/ij/) to semiquantify the expression of N_2_OR. The corresponding histogram shows yield of recombinant N_2_OR in µg N_2_OR/100 µg crude root protein. Recombinant protein yield varied among rolD-*nosZ* samples, with the lowest *nosZ* yield of 0.032 µg N_2_OR/100 µg crude root protein and the highest 0.079 µg N_2_OR/100 µg crude root protein. On average, the rolD-*nosZ* plants expressed N_2_OR at levels 10-fold higher than rolD-*nosFLZDY* plants, which varied from 0.003 µg N_2_OR/100 µg crude root protein to 0.009 µg N_2_OR/100 µg crude root protein.

### Activity of N_2_OR in root tissue

**Figure 3:**
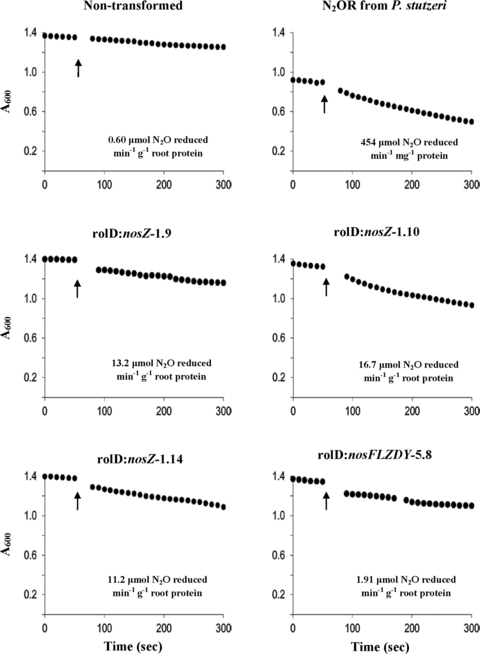
The specific activity of N_2_OR extracted from transgenic root tissue measured by the methyl viologen-linked assay. Trace plots monitor 600 nm absorbance as a function of time. The arrows depict when N_2_O-saturated water was added as enzyme substrate. The immediate sharp but minor drop in absorbance is due to dilution of the reaction mixture in the cuvette. The change in slope after N_2_O addition was used to calculate the specific activities shown above each curve.

The specific activity of N_2_OR in four transgenic tobacco lines was assessed using a spectrophotometric methyl viologen-linked assay. A_600_ was plotted against time for the slope-based activity calculations, and the calculated specific activity of the recombinant enzyme (expressed as µmol N_2_O reduced min^−1^ g^−1^ root protein) is presented in Fig. 3. The nontransformed control showed minimal activity (0.60 µmol N_2_O reduced min^−1^ g^−1^ root protein). The positive control, N_2_OR from *P. stutzeri*, gave a specific activity of 454 µmol N_2_O reduced min^−1^ mg^−1^ protein. N_2_OR from the four samples assayed showed considerable variability in their specific activity. Activity was assayed for one individual plant per transgenic line, for the same lines assayed in Figs. 1 and 2.

### Protein expression analysis of rhizosecreted N_2_OR

**Figure 4:**
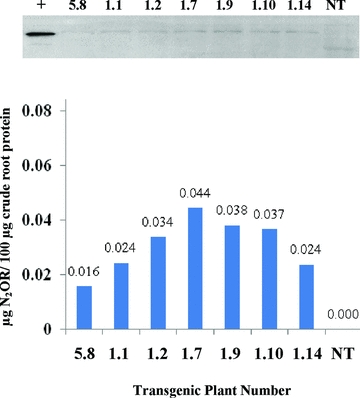
Western immunoblot analysis detecting N_2_OR in the rhizosphere surrounding transgenic plant root tissue. Histogram showing the yield of recombinant N_2_OR in *nosZ*-expressing and *nosFLZDY*-expressing transgenic plants, with the corresponding Western immunoblot. + = positive control, N_2_OR protein purified from *P. stutzeri*; NT = negative control, protein extracted from a nontransformed plant; transformed plants are identified by number.

A Western immunoblot was performed to test for the expression and subsequent excretion of recombinant N_2_OR into the rhizosphere. Fractions of hydroponic medium collected over a 25-day period from individual plants were pooled, and the crude protein extracted. The resulting immunoblot is shown in Fig. 4. A 72-kDa band coinciding with the size of the protein from *P. stutzeri* is present in the exudates from all transgenic lines. The nontransformed control did not exhibit a signal for the presence of N_2_OR. Yield of recombinant N_2_OR was determined based on the chemiluminescent signal and ranged from 0.016 µg N_2_OR/100 µg crude root secreted protein (rolD:*nosZ*-5.8) to 0.044 µg N_2_OR/100 µg crude root secreted protein (rolD:*nosZ*-1.7).

### Activity of N_2_OR in rhizosphere

**Figure 5:**
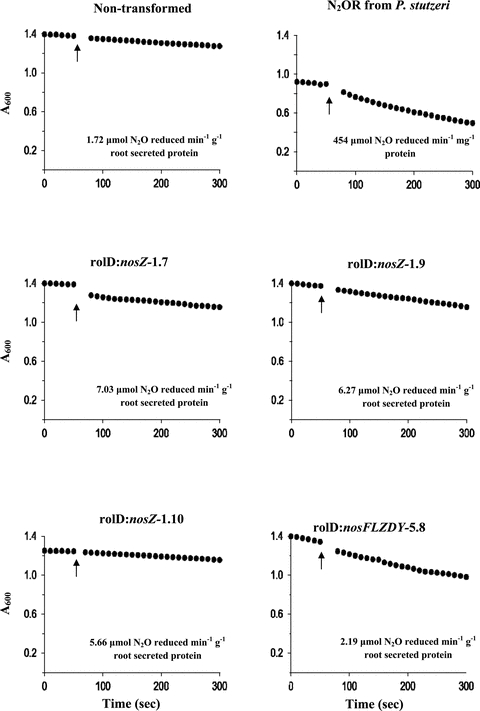
The specific activity of transgenic plant root-excreted N_2_OR in the rhizosphere as measured by the methyl viologen-linked assay. Trace plots monitored 600 nm absorbance as a function of time. The arrows depict when N_2_O-saturated water, the enzyme substrate, was added as enzyme substrate. The immediate sharp but minor drop in absorbance is due to dilution of the reaction mixture in the cuvette. The change in slope after N_2_O addition was used to calculate the specific activities shown above each curve.

The crude protein extracted from the hydroponic culture medium was assayed for specific activity using the methyl viologen-linked assay. Fig. 5 reports the calculated specific activity of the recombinant enzyme, expressed as µmol N_2_O reduced min^−1^ g^−1^ root secreted protein. The nontransformed control showed a background specific activity (1.72 µmol N_2_O reduced min^−1^ g^−1^ root secreted protein). The purified enzyme itself serving as a positive control, N_2_OR from *P. stutzeri*, gave a specific activity of 454 µmol N_2_O reduced min^−1^ mg^−1^ protein. N_2_OR from the rolD:*nosZ*-1.7, rolD:*nosZ-*1.9, and rolD:*nosZ-*1.10 transgenic lines showed the highest specific activities of 7.03, 6.27, and 5.66 µmol N_2_O reduced min^−1^ g^−1^ root secreted protein, respectively. This is less than half the specific activities of N_2_OR located in the root tissue of the same transgenic lines (see Fig. 3). In contrast, the specific activity of N_2_OR extracted from the hydroponic media of rolD:*nosFLZDY*-5.8 plants was slightly higher than N_2_OR compartmentalized in the roots (compare 2.19 µmol N_2_O reduced min^−1^ g^−1^ root secreted protein vs. 1.91 µmol N_2_O reduced min^−1^ g^−1^ root protein**)**.

## Discussion

Human-induced climate change has become one of the defining issues of our time given the immense environmental, social, and economic consequences of our continued production and release of greenhouse gases (Callendar 1938). N_2_O emission rates have been exacerbated with the increasing use of synthetic nitrogen-based fertilizers whereby annual N_2_O emissions from global soils now exceed 2.6 Tg (1 Tg = 1 million tons) of N_2_O-N (Davidson 2009).

This study presents a proof-of-concept for the use of N_2_OR-expressing tobacco plants for the phytoremediation of the environmental pollutant N_2_O. We produced expression cassettes for both the *nosZ* gene and *nosFLZDY* genes. The root-specific promoter, *rolD*, provided sufficient expression in tobacco roots. Our findings suggest that *nosZ* gene expression cassettes, both the single cassette prolD:*nosZ* and the megacassette prolD:*nosFLZDY*, were correctly integrated into the tobacco genome and expressed (Fig. 1). Transgenic lines differentially expressed rolD:*FLZDY* (Fig. 1B). This is presumably because the transgene is inserted into the plant genome at random, and expression varies depending on the sequences surrounding the integration site (Clark et al. 1993).

In calculating recombinant protein yield from these transgenic plant roots, two patterns became evident. First, the yield is positively correlated to rolD:*nosZ* expression levels determined by RT-PCR. For example, plant line 1.10 has very strong transcription despite the apparent low content of the transgene (Fig. 1A). This 1.10 mRNA is correspondingly translated into the most abundant level of protein detected by anti-N_2_OR antibodies by western hybridization (Fig. 2A). Plant line 1.10 extracts correspondingly contained the highest amount of protein, 0.079 µg N_2_OR/100 µg root protein. And finally, this plant line 1.10 had the highest specific enzyme activity, 16.7 µmol N_2_O reduced min^−1^ g^−1^ root protein (Fig. 3). The second pattern is the higher N_2_OR expression in rolD:*nosZ* relative to rolD:*nosFLZDY* transgenics. Plants expressing rolD:*nosZ* yielded 10-fold more N_2_OR than those expressing rolD:*nosFLZDY*. A possible explanation for the lower expressing rolD:*nosFLZDY* plants is gene silencing. Since gene silencing increases with increasing transcript size (Johnston et al. 2011; Melnyk et al. 2011), the larger rolD:*nosFLZDY* construct may have been subject to gene silencing in the tobacco plant, whereas the rolD:*nosZ* construct was not.

N_2_OR from rolD:*nosZ* and rolD:*nosFLZDY* plant root tissue was shown to be correctly assembled. Western blot analysis showed, by electrophoretic mobility, that N_2_OR purified from *P. stutzeri* and N_2_OR in crude protein extract isolated from plant root tissue were the same size (Fig. 2). Likewise, in hydroponic culture experiments, we detected N_2_OR in transgenic root exudates, but in a lower quantity than in the root tissue (Fig. 4). These results contrast those from previous experiments, which revealed more recombinant protein in the hydroponic medium than in the tobacco root tissue (Borisjuk et al. 1999). This is due to inadequate transport of the 140-kDa recombinant N_2_OR dimer from root cells across the epidermis and into the rhizosphere. Increasing the permeability of the root cell wall by supplying the plant with a plant growth regulator such as auxin may facilitate better secretion of N_2_OR (Drake et al. 2009).

The present results demonstrate that functional N_2_OR was expressed in the roots and was secreted into the hydroponic rhizosphere in a form able to catalyze the conversion of N_2_O to N_2_ (Figs. 3 and 5). The specific activity of rhizosecreted N_2_OR was half that of N_2_OR isolated from root tissue. We infer that this loss in activity is due to the aerobic nature of the hydroponic culture, as oxygen can cause the inactivation of N_2_OR (Clays-Josserand et al. 1995).

In both plant roots and root exudates, the specific activity of N_2_OR was less than that of the native enzyme in *P. stutzeri* (Figs. 3 and 5). The lower activity of recombinant protein might be due to oxygen inactivation or low partial pressure of N_2_O. The extent to which denitrification occurs is highly dependent upon O_2_ partial pressure and nitrogen availability (Philippot et al. 2009). Since, in these experiments, transgenic plants were grown in the presence of oxygen and with normal levels of nitrogen, optimal N_2_OR production was not fully realized. Just as bacteria grown in anaerobic conditions with N_2_O most highly express N_2_OR (Madigan et al. 2002), the engineered plants also may express more N_2_OR in anaerobic soil types such as those that are heavy textured or which contain a high percentage of organic material (Inglett et al. 2005). An additional factor is exposure to N_2_O. Experiments on *P. stutzeri* showed that the microorganisms are induced to express N_2_OR when grown in the presence of N_2_O (Matsubara et al. 1982). Thus, increasing the concentration of N_2_O in the greenhouse might increase N_2_OR transcription. If the plants were grown in the presence of denitrifying bacteria (e.g., in a field), roots might be exposed to sufficient levels of N_2_O to induce higher production of recombinant N_2_OR (Richardson et al. 2009).

The present results indicate that transgenic tobacco plants stably expressing the *nosZ* transgene are capable of producing functional recombinant N_2_OR. Further analysis will include examination of the ability of these plants to reduce N_2_O in field trials using various soil types and environments together with single plant canopy enclosures to measure nitrogen flux. Once the reduction of N_2_O by the transgenic plant lines has been proven effective in test plots, studies to improve recombinant enzyme yield, as well as stability and activity of the enzyme will be of interest.

Since “arable lands” produce two-thirds of the world's N_2_O (Otañez and Glantz 2011), field crops are logical candidates for N_2_O remediation trials. Crops can be used to amplify the limited capacity of tilled and fertilized soils to reduce N_2_O emissions. About 30 countries have adopted biotech crops such as maize, soybean, canola, and cotton (ISAAA) that are engineered to protect soil and groundwater by reducing the spraying of insecticides and herbicides. Such gene pyramiding is growing rapidly in genetic engineering, delivering many input and output advantages to producers and consumers, for example, enhanced water-use efficiency, enhanced cold tolerance, and increased yield. Extending the remit of biotech crops to include soil gas flux, namely, reduction of fertilizer-induced N_2_O emission, should encounter positive regulatory approval given the potential contribution to purge those same soils of this deleterious gas (Ghimire et al. 2011; Wan et al. 2011). Agricultural soils emit 40 g N_2_O ha^−1^ day^−1^ (S. Strand, University of Washington, pers. comm.). If N_2_OR was expressed in maize, the conversion of N_2_O by the roots of maize could be as high as 2.11 × 10^3^ g N_2_O ha^−1^ day^−1^ (Supplementary Methods), more than 50 times greater than N_2_O emissions from agricultural soil. By accumulating sufficient levels of N_2_OR, soils growing these transgenic crops could capture and alleviate N_2_O pollution.
